# Gut microbiota, allergic rhinitis, vasomotor rhinitis, Mendelian randomization, causal association

**DOI:** 10.1016/j.bjorl.2024.101491

**Published:** 2024-08-12

**Authors:** Xitan Lin, Xiaoyan Hu, Jing Zhang, Jing Luo, Gang Qin, Liang Jiang

**Affiliations:** a Affiliated Hospital of Southwest Medical University, Department of Otolaryngology Head and Neck Surgery, Sichuan, China; b School of Basic Medicine, Department of Pathogen Biology, Southwest Medical University, Public Center of Experimental Technology of Pathogen Biology Technology Platform, Sichuan, China

**Keywords:** Gut microbiota, Allergic rhinitis, Vasomotor rhinitis, Mendelian randomization, Causal association

## Abstract

**Objective:**

Continuous research on the structure and function of intestinal microecology has confirmed the association between gut microbiota and the occurrence, development, and outcome of allergic diseases. Here, we explored the genetic causality between gut microbiota and rhinitis.

**Methods:**

We conducted a two-sample Mendelian Randomization (MR) study to investigate the genetic causal relationship between gut microbiota and allergic rhinitis and vasomotor rhinitis. Genetic variations in the human gut microbiota were obtained from the summary statistics of the MiBioGen study. Genome-wide summary statistics of rhinitis were obtained from the FinnGen consortium. The causal effect between gut microbiota and rhinitis was assessed using the inverse variance weighted, MR-Egger regression, and weighted median methods. In addition, sensitivity analyses were conducted using different methods, including maximum likelihood, simple mode, and weighted model methods.

**Results:**

The IVW approach revealed a causal association of the genus *Ruminococcus gauvreauii* group with an increased risk of allergic rhinitis (IVW Odds Ratio [OR = 1.26] [1.04, 1.53], *p*-value = 0.01645). In addition, the genus *Fusicatenibacter* (IVW OR = 1.20 [1.02, 1.41], *p*-value = 0.02868) was causally associated with an increased risk of vasomotor rhinitis.

**Conclusion:**

Gut microbiota belonging to different genera exert different effects on allergic rhinitis and vasomotor rhinitis, including reducing the risk of rhinitis, and increasing the risk of rhinitis. New insights into the mechanisms of underlying gut microbiota-associated rhinitis are provided.

**Level of evidence:**

Level 5.

## Highlights

• Intestinal flora reduces rhinitis symptoms by reducing pro-inflammatory factors.
• The gut flora can produce neurotransmitters that cause vasomotor rhinitis.
• Keeping the good bacteria in your gut stable can improve the symptoms of rhinitis.
• Bacteria capable of producing short-chain fatty acids (SCFAs) may increase the risk of rhinitis.

## Introduction

Rhinitis^1^ is clinically divided into Allergic Rhinitis (AR) and non-Allergic Rhinitis (non-AR), primarily based on whether it is mediated by allergen-specific IgE. Allergic rhinitis is a non-infectious chronic inflammatory disease of the nasal mucosa that is largely mediated by IgE after the body is exposed to allergens. Vasomotor rhinitis is the most common type of non-allergic rhinitis. Both types are characterized by paroxysmal sneezing, watery rhinorrhea, nasal itching, and nasal congestion. In addition, AR can be accompanied by eye symptoms, including eye itching, tearing, red eyes, and burning sensation. The literature reports that 40% of AR patients could be complicated by bronchial asthma. Nasal symptoms can be accompanied by pulmonary symptoms such as wheezing, coughing, shortness of breath, and chest tightness. Numerous national and international epidemiological surveys have demonstrated a significantly increased prevalence of rhinitis among Chinese individuals in recent years, and AR has emerged as a major chronic inflammatory disease of the respiratory tract, seriously affecting patients’ quality of life and socioeconomics. Research has demonstrated that the onset of rhinitis is related to the interaction of genetics and environment. Although rhinitis has genetic susceptibility, gut microbiota has been implicated in the pathogenesis of allergic rhinitis.

Gut microbiota is a complex community of microorganisms located in the gastrointestinal tract and is critical for maintaining metabolic and immune health.^2^ Clinical studies have reported that patients with lung diseases such as asthma and chronic obstructive pulmonary disease are often accompanied by gastrointestinal diseases such as inflammatory bowel disease or irritable bowel syndrome.^3^ Nearly 50% of adult patients with Inflammatory Bowel Disease (IBD) have lung inflammation or declined lung function. Therefore, modern medicine proposes the concept of “lung-intestine” axis.^4^ Respiratory tract immunity is a component of mucosal immunity due to similar physiological characteristics. The mucosal barrier of the respiratory tract is the first part of the body to come into contact with inhaled antigens such as viruses, bacteria, and allergens.^5^ The intestine, as the largest immune organ in the body, can pass through the common mucosal immune system to affect respiratory immunity.^6^ Gut microbiota affects the respiratory tract through mucosal immunity and is a major factor in driving the development of an individual’s immune system and maintaining immune balance after birth.

Recently, Mendelian Randomization (MR) has been widely applied to evaluate potential causal relationships between exposure factors and outcome variables.^7^ It uses the most common Single Nucleotide Polymorphism (SNP) among genetic variations as the Instrumental Variable (IV) to imitate randomized clinical controlled trials.^8^ Currently, no MR studies are available on the causal relationship between rhinitis and gut microbiota. Therefore, we applied two-sample MR to analyze the causal association between rhinitis and gut microbiota.

## Data source description Methods

### Genetic instrumental variables for gut microbiota

We obtained genetic variations of the human gut microbiota from the summary statistics of the MiBioGen study,^9^ which is the largest cohort and multi-ethnic genome-wide meta-analysis of the gut microbiome conducted to date. The MiBioGen consortium enlisted 18,340 participants from 24 cohorts, encompassing diverse ancestries such as European, American Hispanic/Latin, and East Asian, among others. In total, 211 taxa, including 131 genera, 35 families, 20 orders, 16 classes, and 9 phyla were examined. To ensure specificity, we excluded 15 bacterial traits lacking specific species names, such as those with unknown family or genus information. Finally, 196 bacterial traits were used for further analysis. The microbial composition was assessed using three variable regions (V1‒V2, V3‒V4, and V4) of the 16S rRNA gene.

### Genetic instrumental variables for allergic and vasomotor rhinitis

We obtained the summary statistics from a meta-analysis of a Genome-Wide Association Study (GWAS) on migraine from the FinnGen consortium (https://r4.finngen.fi/). Allergic rhinitis includes participants diagnosed with ICD-10 codes J301, J302, J303, J304, and ICD-9 code 477. However, vasomotor rhinitis includes participants diagnosed with ICD-10 code J300. We performed separate analyses on different subtypes of rhinitis, namely allergic rhinitis (5,527 cases and 212,387 controls) and vasomotor rhinitis (947 cases and 216,195 controls), utilizing publicly available GWAS data (Table 1).

**Table 1:** GWAS summary statistics of allergic and vasomotor rhinitis.

Variable	Number of SNPs	Number of SNPs (Case/Control)	Population	Year
Allergic rhinitis	16380461	217914 (5527/212387)	European	2021
Vasomotor rhinitis	16380465	217142 (947/216195)	European	2021

## Methods

### Mendelian randomization design

**Fig. 1:**
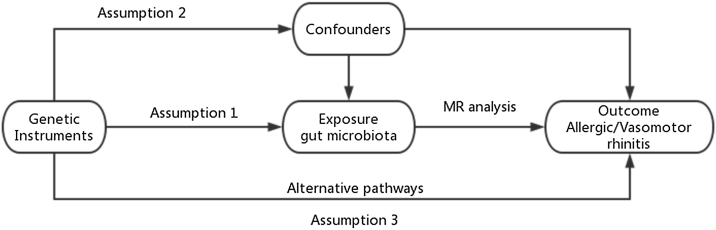
The basic principles of two-sample Mendelian randomization.

The genetic causal relationship between gut microbiota and rhinitis was explored using a two-way MR study approach. Intestinal microorganisms were considered the exposure factor and rhinitis was the outcome variable. The research design is depicted in Fig. 1. The use of these assumptions ensured the reliability and validity of the MR analysis in investigating the causal relationship between gut microbiota and rhinitis.^10^

### Selection of instrumental variable

We implemented a series of quality control measures to select genetic predictors strongly associated with both gut microbiota and rhinitis. This ensured the accuracy of estimating the causal effect of enteric microbiota on rhinitis. Firstly, we applied a stringent genome-wide significance threshold of *p* < 5 × 10^−8^ to identify SNPs exhibiting strong associations with both gut microbiota and rhinitis. However, we adopted a relatively comprehensive threshold of *p* < 1 × 10^−5^ to include a wider range of potentially relevant SNPs due to the limited number of eligible Instrumental Variables (IVs). To satisfy the assumptions required for the MR analysis, we conducted a Linkage Disequilibrium (LD) analysis based on the European-based 1,000 Genomes Project. We set an R2 threshold of less than 0.001 and a clumping distance of 10,000 kb to account for LD between SNPs. SNPs not meeting these requirements were excluded from the analysis. In addition, we excluded palindromic SNPs due to uncertainty about their alignment in the same direction for both exposure and outcome in the GWASs of rhinitis. These quality control steps were implemented to ensure the validity and reliability of instrumental variables used in the MR analysis investigating the causal relationship between enteric microbiota and rhinitis.

### Main analysis

We used the Inverse-Variance Weighted (IVW) method as the primary analysis to estimate the causal effect of intestinal flora on rhinitis.^11^

### Additional analysis

We used certain additional analysis methods, including the maximum likelihood method, MR-Egger regression, weighted median method, simple model, and weighted model methods to evaluate the causal effect of intestinal flora on rhinitis. The IVW method was considered positive in cases where it yielded a significant result (*p* < 0.05), even if the other methods did not reach significance, as long as the beta values of other methods were consistent in direction.

Statistical analysis was performed using the “TwoSampleMR”, “MRInstruments” and “Mendelian Randomization” software packages in R4.1.3 software, with a test level of α = 0.05. A priori statistical power is calculated using the non-centrality parameter of the test statistic proposed by Brion et al. (http://cnsgenomics.com/shiny/窗体底端).

## Results

### Weak instrumental variables test

A total of 196 bacterial taxa, comprising 9 phyla, 16 classes, 20 orders, 32 families, and 119 genera, were considered for the instrumental variable analysis. Following a meticulous process of instrument selection, the number of SNPs associated with each bacterial taxon varied from 6 to 18. Importantly, all F-statistics exceeded 10, indicating the absence of weak instrumental variables (Table 2).

**Table 2:** A summary of GWAS summary statistics for different risk factors.

Exposure	NSNP	Sample size	R2%	F
Family *Lactobacillaceae*	11	14306	0.15	22.80
Genus *Eubacterium brachy group*	18	14306	0.16	24.24
Genus *Fusicatenibacter*	15	14306	0.16	24.23
Genus *Prevotella9*	11	14306	0.20	29.12
Genus *Ruminococcaceae* UCG004	10	14306	0.15	21.66
Genus *Ruminococcaceae* UCG010	6	14306	0, 15	22.25
Genus *Subdoligranulum*	8	14306	0.17	24.55

### Main analysis results

**Fig. 2:**
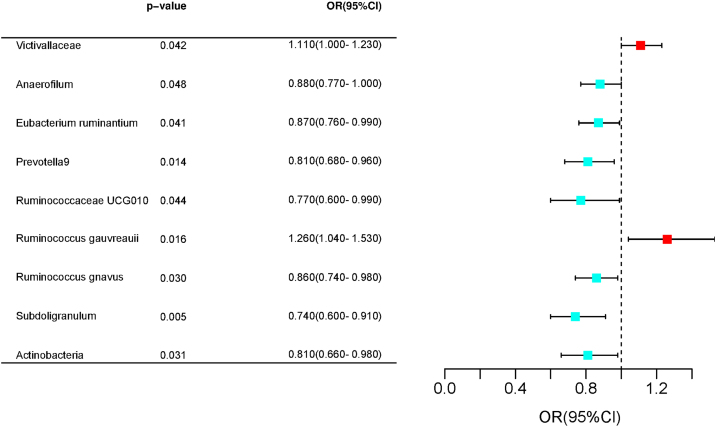
Forrest plot for summary causal effects of gut microbiota on allergic rhinitis risk based on IVW method for the primary analysis.

**Fig. 3:**
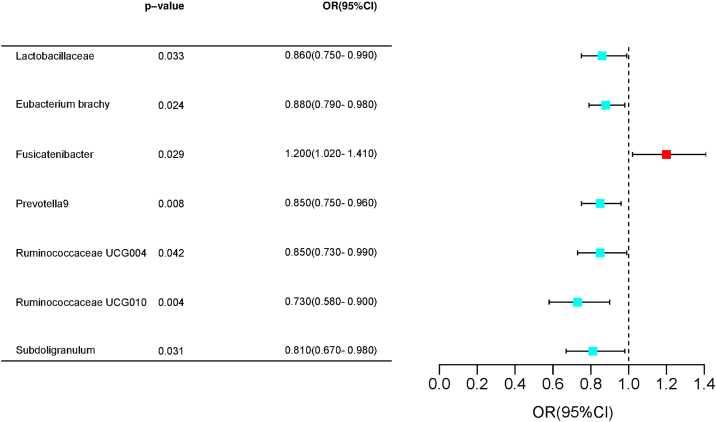
Forrest plot for summary causal effects of gut microbiota on vasomotor rhinitis risk based on IVW method for the primary analysis.

The IVW approach demonstrated that the family *Victivallaceae* (OR = 1.11, 95% CI 1.00–1.23, *p* = 0.04176) and genus *Ruminococcus gauvreauii* group (OR = 1.26, 95% CI 1.04–1.53, *p* = 0.01645) were causally associated with an increased risk of allergic rhinitis, whereas the genus *Anaerofilum* (OR = 0.88, 95% CI 0.77–1.00, *p* = 0.0478), genus *Eubacterium ruminantium* group (OR = 0.87, 95% CI 0.76–0.99, *p* = 0.04117), genus *Prevotella9* (OR = 0.81, 95% CI 0.68–0.96, *p* = 0.01445), genus *Ruminococcaceae* UCG010 (OR = 0.77, 95% CI 0.60–0.99, *p* = 0.04417), genus *Ruminococcus gnavus* group (OR = 0.86, 95% CI 0.74–0.98, *p* = 0.02961), genus *Subdoligranulum* (OR = 0.74, 95% CI 0.60–0.91, *p* = 0.00489), and phylum Actinobacteria (OR = 0.81, 95% CI 0.66–0.98, *p* = 0.03055) were causally associated with decreased risk of allergic rhinitis. In addition, we found the genus *Fusicatenibacter* (OR = 1.20, 95% family *Lactobacillaceae* (OR = 0.86, 95% CI 0.75–0.99, *p* = 0.03316), genus *Eubacterium brachy* group (OR = 0.88, 95% CI 0.79–0.98, *p* = 0.02364), genus *Prevotella*9 (OR = 0.85, 95% CI 0.75–0.96, *p* = 0.00778), genus *Ruminococcaceae* UCG004 (OR = 0.85, 95% CI 0.73–0.99, *p* = 0.04249), genus *Ruminococcaceae* UCG010 (OR = 0.73, 95% CI 0.58–0.90, *p* = 0.04248), and genus *Subdoligranulum* (OR = 0.81, 95% CI 0.67–0.98, *p* = 0.03087) to be causally associated with a decreased risk of vasomotor rhinitis. These results are presented in Fig. 2, Fig. 3.

### Additional analysis results

The results obtained from both MR-Egger and WME methods were consistent with IVW results.

### Sensitivity analyses

We first applied the leave-one-out method to eliminate each SNP one by one, followed by the IVW method to conduct an effect analysis on the remaining SNPs. No SNP sites in instrumental variables exerted a strong impact on the results, indicating the robustness of the research results.

## Discussion

We used the MR research methods to systematically evaluate the causal relationship between intestinal flora and the risk of two types of rhinitis. Three types of bacterial flora were related to the reduction in the risk of two types of rhinitis.

Microbiota living in the human digestive system are a vital component of human life and have been implicated in the organization and performance of the human body.^12^ Dendritic Cells (DC) in the nasal mucosa absorb allergens and release inflammatory mediators using a series of immune responses, ultimately leading to the development of allergic rhinitis. The association between gut health and allergies begins with the immune system. All components of the immune system are directly or indirectly regulated by the microbiome, and studies have demonstrated that the immunomodulatory effect of the gut microflora on allergic rhinitis depends on the influence of the intestinal cytokine microenvironment on the immune response of the distal respiratory tract.^13^ Vasomotor rhinitis is non-allergic rhinitis, which is currently believed to be largely related to autonomic nervous system dysfunction and non-IGE-mediated inflammatory response. For example, certain neuropeptides released through the axon reflex will not only amplify the stimulation signal but also lead to increased vascular permeability, hypersecretion of glands, and even induce mast cells to degranulate and release histamine, causing anaphylactic reactions.^14^ The autonomic nervous system exists between the intestinal flora and the nervous system, whereas the intestinal flora affects the function of the nervous system by decomposing food and producing metabolites. The gut microbiota produces different hormones and neurotransmitters, which can affect the regulation of the nervous system, resulting in the development of vasomotor rhinitis.

We found nine gut microbiotas with a nominal causal relationship with AR. Seven groups of bacteria were protective against AR, including the genus *Anaerofilum*, genus *Eubacterium ruminantium* group, genus *Prevotella9*, genus *Ruminococcaceae* UCG010, genus *Ruminococcus gnavus* group, genus *Subdoligranulum*, phylum *Actinobacteria*. *Actinobacteria* constitute a diverse group of gram-positive bacteria. Although Actinobacteria represent only a small fraction, they are involved in maintaining intestinal homeostasis. Almost all Actinobacteria are involved in microbial homeostasis; certain probiotics and others as pathogens causing inflammation. The most outstanding characteristic of actinomycetes is that they can produce a wide variety of antibiotics in abundance.^15^ Among them, bifidobacteria in actinomycetes represent a beneficial bacteria group, because it can synthesize B vitamins, thus promoting gastrointestinal peristalsis and improving constipation. Recently, clinical trials have reported that it can reduce nasal mucosal edema, thereby alleviating nasal congestion, runny nose, and other symptoms caused by rhinitis. Relevant studies have proved that oral bifidobacteria intervention in early life can effectively inhibit the symptoms of allergic rhinitis in mice later in life, which could be attributed to the inhibition of Th2 cell response and enhancement of the function of CD4^+^CD25^+^Treg cells.^16^ Certain microorganisms, including bifidobacterium, are known to positively impact intestinal health and immunity by promoting the growth of beneficial bacteria and reducing the number of harmful bacteria, thereby reducing the risk of rhinitis or alleviating the symptoms of rhinitis.^17^

In total, two positive causal relationships were identified, including the family *Victivallaceae* and genus *Ruminococcus gauvreauii* group with AR. *Ruminococcus* is a gram-positive anaerobic bacterium belonging to the family *Peptoeaccaeeae*. *Ruminococcus* is one of the first gastric bacteria discovered and contributes to metabolism.^18^ Relevant studies have demonstrated that rumen cocci can stabilize the intestinal barrier, reverse diarrhea, and reduce the risk of colorectal cancer and kidney stones. In addition, it is associated with intestinal diseases (IBS, IBD, Crohn’s disease, etc.), immune diseases (allergy, eczema, asthma, etc.), neurological diseases (autonomous diseases, etc.), autism, depression, etc.). *Ruminococcus* is roughly divided into two types, namely *Ruminococcus albus* and *Ruminococcus gnavus*.^19^ Rumen coccal polysaccharides can stimulate immune system cells, such as Immunoglobulin E (IgE) and Tumor Necrosis Factor (TNF)-α, which are inflammatory biomarkers of AR symptoms.^20^ Glucorhamnan, a complex polysaccharide with rhamnose-skeleton and glucose side chain, is synthesized and secreted by *Ruminococcus* active, which can effectively induce dendritic cells to secrete inflammatory cytokines (TNF-α).^21^ It is speculated that if the gene corresponding to this polysaccharide secreted by rumen cocci is expressed before rhinitis worsens, it proves this polysaccharide to be the cause of rhinitis. This information could be used to develop new treatments for rhinitis and similar inflammatory diseases based on rumen coccus and its secreted polysaccharides.

In addition, we found seven gut microbiotas nominally and causally associated with vasomotor rhinitis. Genus *Eubacterium brachy* group, genus *Prevotella9*, genus *Ruminococcaceae* UCG004, genus *Ruminococcaceae* UCG010, and genus *Subdoligranulum* belonging to the family *Lactobacillaceae* were causally associated with a decreased risk of vasomotor rhinitis. *Lactobacillaceae* has a relatively large proportion. Lactobacilli not only improves the digestion and absorption of nutrients and maintains the balance of intestinal flora, but also regulates immune responses. Several species of *Lactobacillus* produce Gamma-Aminobutyric Acid (GABA), a major inhibitory neurotransmitter in the brain. Mental tension, anxiety, environmental temperature change, and endocrine dysfunction can cause excessive release of parasympathetic neurotransmitters, resulting in the non-specific release of histamine, vasodilation, increased glandular secretion, and corresponding clinical symptoms, and finally vasomotor rhinitis. GABA produced by *Lactobacillus* can effectively reduce the incidence of vasomotor rhinitis. *Lactobacillus* alleviates the inflammatory response by inter-regulating pro-inflammatory and anti-inflammatory cytokine responses, thereby alleviating the clinical symptoms of vasomotor rhinitis.

Studies have shown that the genus *Fusicatenibacter* is positively associated with the risk of developing vasomotor rhinitis. Studies have demonstrated that *Fusicatenibacter* bacteria are intricately related to inflammatory diseases.^22^ *Fusicatenibacter* is a species of the genus *Clostridium* and can produce Short-Chain Fatty Acids (SCFAs). SCFAs produced by intestinal flora can directly regulate host health. The microbial-gut-brain axis is an integral component of the nervous system development. SCFAs can penetrate the blood-brain barrier, transfer to the brain through blood circulation, and affect the autonomic nervous system or immune system and inflammatory response. This suggests that disturbed microbial metabolism can greatly impact vasomotor rhinitis with autonomic nervous dysfunction. Therefore, we can assume that the progression of vasomotor rhinitis can be predicted by detecting changes in SCFA-producing bacteria.

Intestinal flora can prevent the occurrence and development of allergic or vasomotor rhinitis by reducing serum pro-inflammatory factors, increasing the number of immune cells, regulating the balance of Th1 and Th2, elevating the number of Tregs, and regulating autonomic nervous functions. Increased levels of beneficial bacteria can regulate the stability of gut microbiota, restore the intestinal mucosal barrier, maintain autonomic nervous function, and thus improve rhinitis.

This study has several strengths. This was the first MR analysis using two samples to investigate the possible causal connection between gut microbiota and rhinitis. Traditional observational studies are more prone to the potential for bias due to the presence of confounding variables and the possibility of reverse causality. Next, the summary-level data on gut microbiota comprised the largest Genome-Wide Association Study (GWAS) to date, and the dataset was based on multiple human populations. This allowed our findings to be generalized to different human groups. In addition, MR analysis is a continuously growing technique whose epidemiological impact is enormous. As more genetic data become available and new methods are developed, MR analysis will continue to be a valuable tool for understanding the causal relationship between risk factors and disease outcomes.

Still, there are limits to what can be drawn from this study. First, there is a lack of basic demographic information and clinical presentation data. This limitation prevents us from further studying the causal relationship between gut microbiota and rhinitis at the species level. Second, the lowest classification level in the exposure data is the genus, which hinders our study of relationships at the species level. Third, different risk factors may influence the risk of allergic rhinitis and vasodilator rhinitis ‒ immune and clinical/functional outcomes are also important based on age and sex, but we did not consider them due to data modelling. Fourth, lifestyle factors and environmental exposures that influence gut microbiome composition and rhinitis risk were not controlled for in this analysis. Finally, although we conducted an extensive literature review and identified a number of confounding factors, there may still be potential unknown confounding factors that could have an impact on the results. Therefore, more care should be taken in interpreting the results.

## Conclusion

In conclusion, we performed a two-sample MR analysis using publicly available GWAS summary-level data to assess the causal impact of gut microbiota on rhinitis and identify potentially pathogenic flora for the development of rhinitis. This study could be useful for screening gut microbial-based metabolites and markers for the early detection of rhinitis as non-invasive diagnostic or therapeutic targets.

## Authors’ contributions

Xiaoyan Hu and Xitan Lin contributed equally to this study.

## Funding

This study was supported by Sichuan Science and Technology Program (2022YFS0629, 2023YFS0090) and Joint Fund of HeJiang and Southwest Medical University (2022HJXNYD06).

## Ethical statement

This article was approved by the Ethics Committee of the Affiliated Hospital of Southwest Medical University. We confirm that the manuscript containing any individual person’s data received consent from the patients and their families for publication.

## Conflicts of interest

The authors declare that the research was conducted in the absence of any commercial or financial relationships that could be construed as a potential conflict of interest.
